# Local Violence as an Environmental Exposure with Increasing Relevance under Climate Change: A Conceptual Framework

**DOI:** 10.1007/s11524-026-01071-5

**Published:** 2026-06-02

**Authors:** Jane E. Clougherty, Ellen J. Kinnee, Perry E. Sheffield

**Affiliations:** 1 Department of Environmental and Occupational Health, Drexel University Dornsife School of Public Health, Philadelphia, USA; 2 University Center for Social and Urban Research (UCSUR), University of Pittsburgh, Pittsburgh, USA; 3 Department of Pediatrics, Icahn School of Medicine at Mount Sinai, New York, USA; 4 Department of Environmental Medicine, Icahn School of Medicine at Mount Sinai, New York, USA; 5 Urban Health Collaborative (UHC), Drexel University Dornsife School of Public Health, Philadelphia, USA

**Keywords:** Violence, Community health, Climate change, Psychosocial stress

## Abstract

Violent crime is increasing worldwide with climate change and ambient heat, with greater increases projected in communities already high in crime or lacking resources for mitigation, exacerbating inequities in exposures and health. Our work has shown that violent crime—as a severe psychosocial stressor—can exacerbate effects of climate-related heat and pollution on health. In addition, as heat is associated with greater violence, and both heat and violence are shown to negatively influence child mental and physical health, some proportion of heat impacts on health may plausibly be mediated through local violence. Taken together, there is a need for frameworks and methods to disentangle the independent and synergistic effects of climate-related violence, heat, and pollution on child health, and to translate this understanding into clinical and public health action to protect patients and families. Here, we propose a framework for examining violence as a climate-related environmental exposure, and discuss (1) conceptualization of violence as an environmental variable, (2) impacts of violent crime on perceived stress, (3) measuring local violence for epidemiology, (4) pathways for impacts of local violence on health in the context of climate change, in combination with other climate-related exposures, and (5) implications for policy and practice.

## Introduction

Violent crime is increasing with climate change and ambient heat [1–5], though its potential impacts on health have not been emphasized in climate epidemiology to date. Violence currently costs over 19,000 lives annually in the USA, and the firearm-related death rate in the USA is over 11 times higher than in 28 other high-income nations [6]. A 2024 US Surgeon General advisory report stated that firearm violence was the leading cause of death for children and adolescents since 2020, explicitly calling for public health responses [6].

Homicides are expected to increase 6% globally per degree increase in global annual mean temperature, and an additional 2.3–3.2 million heat-attributable violent crimes are expected in the USA from 2020 to 2099 [7]. An analysis of 100 US cities recently attributed 6.85% of shootings (7973 events) to elevated temperatures [1]. Violence may thus account for a significant proportion of heat-related morbidity and mortality. Violence is projected to increase more in locations that are already high in violence or have limited economic resources for response [2], further exacerbating inequities in exposures and health.

**Fig. 1:**
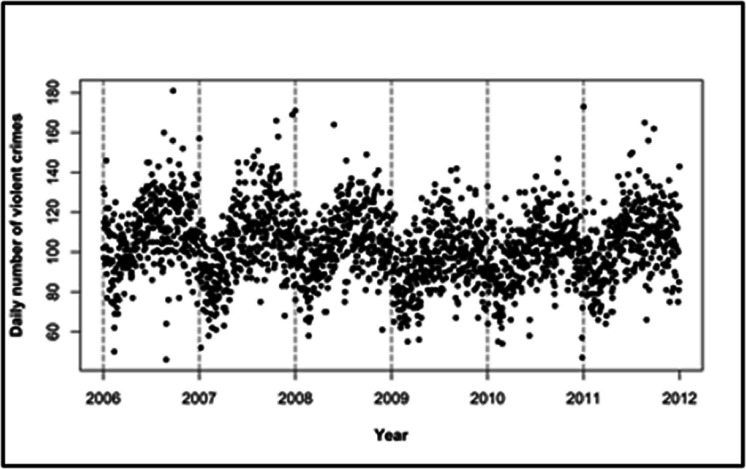
Daily trends in reported violence in NYC over 2006–2023. Violent crime is consistently higher in summer months

Violence in several forms—interpersonal, state, and political violence—is projected to increase with warming temperatures, given direct impacts of heat on aggressivity [8–10], and the pressures of resource scarcity, forced migration, and economic strains expected in many regions of the world [11]. Here, we focus on local interpersonal violence in the immediate community context (as opposed to domestic or other forms of violence), shown to be higher during summer months (Fig. 1), and in hotter regions [3].

Two key theories have been proposed to explain observed associations between ambient heat and interpersonal violence [1, 2, 12]: (1) *temperature-aggression theory* [5, 12] posits that heat directly increases discomfort, impulsivity, and aggression and (2) *routine activity theory* [12] posits that, on warm days, more individuals are outdoors, having more interpersonal interactions, creating opportunity for altercations. Stronger observed associations with heat for violent than non-violent crimes [4] support the first theory [13], which is further supported by studies showing more violent crime in hotter countries [14], and both time-series studies and police departments recognizing higher rates of reported violent crime during summer months [2]. Controlled experiments have also shown more aggressive perceptions and responses in hot rooms [15], including the finding that police officers in training were more likely to draw and fire weapons in an uncomfortably hot room [16], and imaging studies indicate that anger activates regions of the brain associated with thermoregulation [16]. *Routine activity theory*, however, is supported by time-series studies revealing crime spikes on aseasonal days (i.e., comfortably warm days in winter) [17], and by at least one study on severity of physical injury (rather than frequency) of maximum daily temperature and outdoor violent crime [18]. These mechanisms may also be mutually reinforcing, if more interpersonal interactions occur on uncomfortably hot days when aggressivity is heightened.

In this framework, we conceptualize local violence as an environmental exposure exacerbated by climate change, and consider (1) the spatial and environmental dimensions of local violence, (2) impacts of violent crime on perceived stress, (3) issues in measuring local violence for epidemiology, (4) pathways for impacts of local violence on health in the context of climate change, and (5) implications for policy and practice.

## Violence as an Environmental Variable

We conceptualize violence as a spatial, or *environmental*, exposure, as individuals needs be physically present, in space and time, to be at risk of victimization. Hence, we focus on “local” violence—occurring in the immediate vicinity of the residence—to emphasize physical proximity and direct bodily risk, although community-scale measures (e.g., police-precinct annual rates) are more widely available and more commonly used in epidemiology. Violence is further “spatial” in that crime rates can be highly persistent across space; we have found, for example, that tract-level annual rates were remarkably stable year-to-year across New York City (NYC) [19].

Violence is also “spatial” in that each violent event can impact entire communities, beyond direct victims or witnesses [20, 21]. Referred to as the “long reach of violence,” [20] violent events and media coverage can lead to pervasive fear, impacting community members’ mental health, sense of safety [22], and local activity patterns (e.g., walking, transit decisions). Fear of violence and avoidance behaviors re-structure how residents physically negotiate their communities; geography research has long shown that urban residents (esp. women) greatly re-structure their daily lives to minimize risks [23, 24]—particularly after dark, or near places with compromised visibility (e.g., greenspaces, parking structures). It has been shown that children (esp. girls) are commonly geographically restricted in outdoor play due to parental fear of violence [25, 26].

Portrayals of violent crime in the media can further entrench place-based notions of crime, particularly in combination with race- or class-based residential segregation, leading to avoidance and stigmatization. As sociologist Patrick Sharkey explains, “Violence happens to people, but it also happens to places.” [20] To this point, we also note that not all exposures to violence are spatial or localized—for example, many Black Americans experience fear and anger following events of police misconduct against Black Americans in other cities [27]—these impacts based on shared identity are the subject of future research.

## Impacts of Violent Crime on Perceived Stress

How does local violence “get under the skin”? [28] As described above, each violent event impacts not only direct victims and witnesses, but also entire communities, though fear and hypervigilance—or, more broadly, perceived stress. Stress, by definition, is the perception that the challenges in one’s life are greater than one’s resources and abilities to meet them [29]. Stress thereby hinges on *perception*, or *appraisal*; in order for an exposure to operate via psychosocial (stress) pathways to health, an individual must actually perceive it to be “stressful.” [29–31] Because stress is based in perception, individual surveys are considered the gold standard measure, though often infeasible for large epidemiologic populations or administrative health datasets. Thus, researchers must often rely on stressor metrics (e.g., poverty rate) as proxies for perceived stress, with limited opportunity to validate their meaning [29, 32, 33].

Community violence is rarely positively appraised, and has emerged as a paramount stressor in our NYC-based work. We found that community violence (measured as self-reported witnessing, or violent crime counts near the home) was the only stressor of concern raised by every focus group across NYC [34], positively correlated with self-reported perceived stress [30, 35, 36], and consistently modified (exacerbated) effects of heat or pollution on health [35, 37]. We emphasize, however, that, in other settings or populations, other stressors (e.g., poverty rates) may better explain observed variation in perceived stress.

Given the central role of perception in stress measurement, some form of community input and individual-level assessment is necessary to accurately characterize violence *as it relates to perceived stress*. Community partners can and should inform on aspects of local violence metrics, such as:

1. *Is perceived stress plausibly greater among persons living closer to recent violent events?* [i.e., Does the impact of a violent event on perceived stress among local residents vary with distance from a crime, or are other factors potentially more important?]
2. What is the role of structures in the built environment (e.g., street networks, parks, lighting) in contributing to where and when crime occurs, or how safe residents feel? [38].
3. Does the nature of the crime [e.g., gun vs. non-gun, or targeted (i.e., based on identity, or known relationships among those involved) alter how it affects residents? [21].
4. How do the residents define the boundaries of this “neighborhood” (which rarely coincides with census tracts or police precincts) [39].
5. Can residents identify perceived safe/unsafe spaces in the neighborhood (e.g., parks, garages) [23, 24, 26, 40, 41].

Violent crime impacts on perceived stress may vary by individual characteristics (e.g., sex, age, race [42, 43]), by community characteristics (e.g., SEP, increasing/decreasing crime rates), or characteristics of the violent event itself (e.g., gun vs. non-gun; mass shootings vs. targeted event based on individuals’ relationship) [21]. Violent events may also invoke stronger responses among individuals who feel more susceptible overall (e.g., older adults), or who identify more closely with the victim based on race, ethnicity, or other factors [42, 43] (i.e., underscoring the sense that “it could have been me.”). Impacts of violent crime on perceived stress may be compounded by structural factors [44] indicating disenfranchisement, including redlining and race-based residential segregation [45], or lack of trust in local institutions and police practices [46]. Notably, we have also observed divergent effects of structural factors (i.e., greenspace) on objective crime and perceived safety, particularly by gender [47].

As a violent event’s impact on perceived stress and health—influenced by actual rate of occurrence, detection, and classification—may vary by neighborhood characteristics—including co-occurring stressors, such as poverty, segregation, access to resources, chronic crime rates and trends, policing practices, or incarceration rates [21, 48]—many critical questions remain: Is a single crime event more or less impactful in a community with higher chronic crime? Is each event more impactful where it is “surprising” or “unexpected”? Do some communities, sadly, become inured to violence, such that each event is less impactful? Does a changing political context at the local, state, or federal level of government and/or specific policy changes influence these effects? These nuances in the impacts of crime speak directly to inequity and disenfranchisement, key to understanding compounded impacts of local violence and multiple community stressors.

By better understanding residents’ concepts of local crime and safety—and understanding variation in these perceptions within the community (by age, gender, etc.)—we can better understand residents’ cognitive maps related to perceived safety in their neighborhoods [49], and tailor measures to better capture violent crime *as it relates to stress and health*, for epidemiology purposes.

## Measuring Local Violence for Epidemiology

In exposure science and epidemiology, we generally estimate physical exposures (i.e., heat, pollution) at the finest spatial and temporal scale possible (e.g., daily 100 m resolution around residence) [50]. In contrast, we often use coarse administrative metrics for social stressors (e.g., tract-level poverty rates), potentially inducing substantial misclassification [50–52]. We found that, measuring social stressors and pollutant exposures with equal resolution (tract-level annual averages), the former explained far more variability in cardiovascular event rates across NYC [50]. This result suggests that substantial misclassification may be induced by relying on coarse aggregate metrics for social stressors, potentially dampening effect estimates for stressor exposures [53], and biasing estimates for environmental co-exposures. Further, because violent events can be sporadic, with both acute and chronic impacts on perceived stress and health [54], there is a particular need for metrics refined in both space and time.

Criminologists have developed space-time metrics for violent crime as an objective outcome (not as a perceived stressor) to identify its antecedents [55–62], towards reducing occurrence and victimization [57, 59, 63–65]. For example, the Boston Area Research Initiative at Northeastern University has shown vast hyper-localization in violence, with crime in each tract driven by one or a few blocks, and crime on those blocks largely occurring near one or a few addresses [40, 41]. The same has been shown for shootings data in NYC [66]. Thus, spatially coarse metrics (i.e., tract-level annual rates) misclassify violence exposures in most of the tract, and fully ignore seasonal variation and individual variation in perceived risk.

It is different to conceptualize violence as a psychosocial stressor (as in epidemiology) than as an objective outcome (as in criminology), as the former must operate via perceived stress pathways to health, as described above. The spatial and temporal dimensions of the violence-stress relationship have not been well characterized for purposes of epidemiology, and many questions remain:

- Are there critical distances within which residents may be more impacted by violent events?
- Is a violent event on one’s block more impactful than one that occurs two or three blocks away?
- How long do the acute stress impacts last?
- Do these relationships vary by age, race ethnicity [43], gender, or socioeconomic position (SEP)?
- Are perceived risks more closely related to one’s proximity to a crime, or to the spatial density of crimes in the area (i.e., event per km^2^)?
- How does population density affect perceived risks (i.e., Do individuals “discount” perceived risks in more populated areas, where one’s statistical odds of victimization may be reduced, given a larger number of potential targets (i.e., crimes/10,000 daytime or residential population)?
- Is each crime’s marginal impact greater or lesser in areas of high or rising crime rates [67]?

While several studies have examined associations between time-varying crime rates (at the neighborhood or small-area level) with mental distress [67, 68], only two, to our knowledge, have examined spatially refined relationships between violent events and well-being, each only in children, and each testing only a few concentric buffers and timepoints. As such, none of the studies to date, to our knowledge, has been designed to identify critical distances or timelags in these relationships. Vasan et al. found elevated risk of child mental health emergency department visits in Philadelphia following shootings, with stronger effects among children living within ¼-mile (vs. ½-mile), and in the first 14 days (vs. 30 or 60 days) [69]. Sharkey found greater deficits in test scores among children living closer to a crime (within block group), and in the first 4 days [43]; effects did not vary by sex or age, but were stronger among Black children. Noting that most victims were also Black, this latter result suggests that children identifying more closely with the victim may experience greater impacts [43].

Some smaller epidemiologic studies have measured direct witnessing or victimization using surveys [70, 71] time/space diaries, smartphones, GPS devices, or social media to detail activity spaces [55, 72–75], but these methods can lack scalability to larger datasets. Thus larger epidemiologic studies (such as those leveraging electronic medical records) have relied on coarse areal metrics (e.g., precinct-level annual rates) [8, 71, 76, 77] which can obscure the vast micro-variation in violence discussed above [40, 41], and ignore perception and stress entirely.

Taken together, we suggest that, if exposure to local violence is hypothesized to operate via psychosocial (stress) pathways [78, 79], researchers take steps to validate metrics against perceived stress or mental health measures, and examine fine-scale spatial and temporal relationships between violence and stress/health, noting that non-linearity in these relationships may reveal critical distances and time lags for intervention.

## Pathways for Impacts of Local Violence on Health in the Context of Climate Change

Local violence, as a psychosocial stressor, may impact health alone or in combination with other climate-related exposures. Despite this complexity—and the potential importance of exposures to local violence as a growing determinant of health under climate change—studies have not yet begun to disentangle the interplay among violence [35, 37, 70], heat [78, 80, 81], and pollution [37, 82] on health, under climate scenarios.

**Fig. 2:**
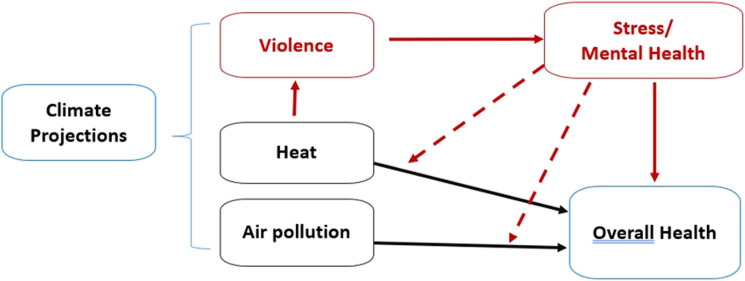
Conceptual model. Solid lines are hypothesized direct or mediation paths; dotted lines indicate effect modification

**Fig. 3:**
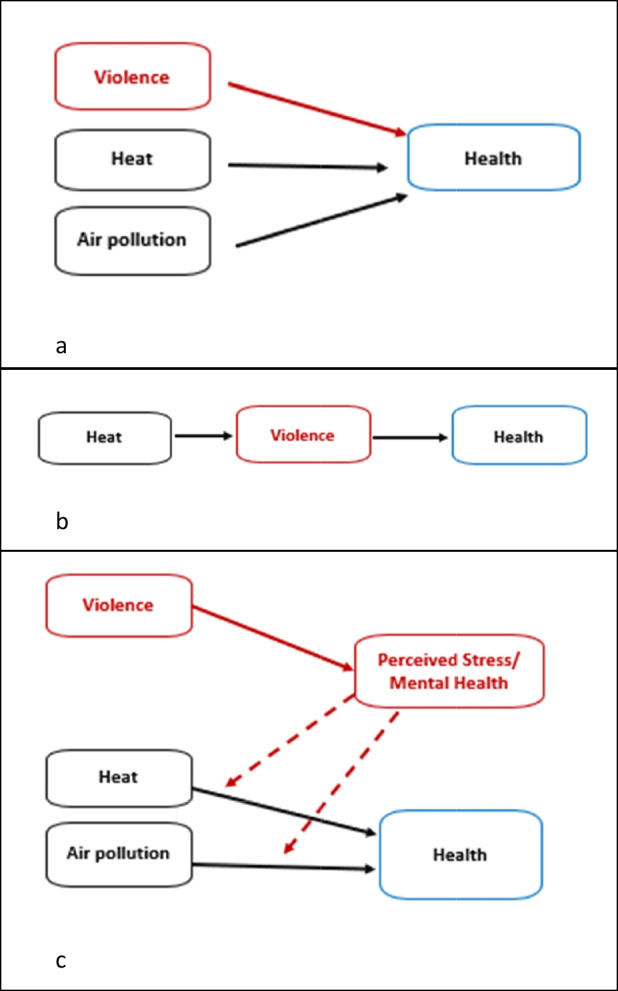
Conceptual models for **a** independent associations for ambient heat, pollution, and local violence on health, **b** impacts of ambient heat on local violence, which, in turn, impact health, and **c** as a chronic stressor, local violence may exacerbate (modify) impacts of heat and pollution on health

In each pathway described here, we conceptualize local violence to operate through psychosocial stress [83] (we do not address direct impacts of victimization in this paper). Our overall conceptual model is presented in Fig. 2, with specific hypothesized pathways discussed below, and shown in Fig. 3a–c.

- **Exposures to** **local violence may directly impact mental and physical health through perceived stress pathways.**

Exposure to local violence and other climate-related exposures may independently increase health risks (Fig. 3a), and the relative impact of each will likely vary substantially by outcome, population, and setting. Few studies have examined associations between refined time-space violence metrics and physical health in urban communities [84], and none, to our knowledge, using climate projections or in the context of climate-related co-exposures. Because violence is higher in summer (Fig. 1), when heat, humidity, and some climate-related air pollutants (i.e., O_3_, PM_2.5_) are also higher, and because heat is associated with higher crime [1], these exposures may be highly confounded, and thus it is likely necessary to account for each in a refined manner.

b) **Higher local violence may account for (mediate) some portion of observed heat-health associations.**

Extreme heat is shown to confer substantial impacts on morbidity and mortality, in children and adults [85, 86].

Increasing heat exposures also confer increases in violent crime [1, 4, 10] and psychological distress [78, 87]. As such, some proportion of heat-attributed impacts on health may be mediated via violent crime and perceived stress. Our field has not yet investigated impacts of climate-related violent crime exposures, refined in space and time, with as much rigor as for heat or pollution, plausibly *under-estimating* the role of local violence along the heat-health pathway (Fig. 3b).

c) **Violence may increase susceptibility to heat and air pollution (effect modification).**

Violence impacts human health via direct (psychosocial) [88, 89] and indirect (behavioral) pathways (e.g., smoking, alcohol consumption) [90, 91], on both chronic and acute timescales. Further, severe and chronic psychosocial stress is shown to compromise immune, endocrine, and metabolic function (collectively referred to as “allostatic load”) [28, 92–95]. Exposure to local violence (and other severe stressors) can thereby render individuals more susceptible to the health effects of air pollution [19, 32, 96, 97], extreme heat [37], and other physical exposures (e.g., viruses) [98] (Fig. 3c). Exposure to chronic psychosocial stressors is shown to exacerbate associations between traffic-related air pollution and asthma etiology and other outcomes among children [70, 99], and alter effects of PM_2.5_ on respiratory function in animal models [100]. In NYC, local violence more strongly modified associations between ozone (O_3_) and child asthma events than did community-level socioeconomic position (SEP) [35, 37]. Because all three of these exposures—air pollution, heat, and violence—are expected to increase with climate change [35, 101], each will bear direct consequences on population health, and attention to the potential for synergistic effect among these is greatly warranted.

## Implications for Policy and Practice

Better understanding the dynamics of local violence and its impacts on perceived stress and health under climate change can inform policy and practice, including urban planning, cumulative risk analyses [102], and clinical practice. Violence is a key challenge in many US cities—with some experiencing increasing gun violence, especially as relates to children’s exposures [102], despite declining violence overall [6], and many cities are now prioritizing gun violence reduction strategies. Violence is currently more prevalent in cities, in the US context (i.e., NYC and Buffalo comprise 61% of violent crimes in NYS, but only ~45% of population), but correlates with stress and health in many settings [36, 103, 104]. Addressing violence and safety in urban planning will become a greater priority as the world population is increasingly urbanizing, and with greater warming in urban areas due to urban heat island effects [105, 106]. Accordingly, some highly localized interventions emphasizing safe urban playspaces [107] have demonstrated reductions in child exposures to both heat and violence in cities, and consequent improvements in child well-being.

Violence-related trauma can have lifelong health impacts [79, 104, 108, 109], and thus expedient, effective responses are critical to reducing exposures and impacts on children, in particular. Improved measurement of stress-related impacts of violence can improve assessment of intervention efficacy, towards reducing violent crime and its impacts, improving responsiveness of municipal services to address community needs and ultimately improving trust, as demonstrated by the National Network for Safe Communities [110]. Such refined measures may also help to quantify the effectiveness of community-engaged interventions including violence interrupter models, which train residents to recognize and disrupt interpersonal conflicts before escalating into violence, as in the Cure Violence approach (formerly Ceasefire) [111], which has become an international model for reducing interpersonal violence in urban communities.

Refined health-relevant exposure to local violence metrics can inform social determinants screeners in clinical settings [112] and health records [113], and help improve mental health (MH) program delivery via early identification. Improved metrics may aid hospitals, emergency departments, first responders, and others involved in responding to violent events. Better understanding the spatial extent of impacts from violent events, and anticipated longevity of impacts, can help clinicians, schools, government, and institutions to better plan for, and deliver, targeted counseling, resources, and trauma-informed care [114], including hospital-based programs as developed by the Health Alliance for Violence Intervention, which provides coordinated care from hospital settings through community-led efforts to reduce violence, promote equity, and ultimately improve community health [115].

In clinical care, a more rigorous inclusion of local violence exposure in screeners [such as those for Adverse Childhood Experience (ACEs) [114, 116–118] now reimbursable via health insurance in some states [119] could improve resource delivery to patients and families. This work can help improve ongoing clinical efforts to integrate environmental exposures into routine primary health care [120, 121]. Despite vast evidence on the importance of social determinants of health [122, 123], clinical intervention has been stymied in lacking one-size-fits-all pharmacologic-style interventions for violence and stressor exposures [124]. As one example, the New York State Children’s Environmental Health Centers (NYSCHECK) developed *Rx for Prevention*s using a screen-counsel-refer approach to help clinicians to “prescribe” local resources [125, 126]. Such tools could help to incorporate messages as the evidence base develops for assessing impacts of exposure to local violence and identifying best practices and recommended action steps.

Detailing the complex role of local violence in health—and prioritizing gun violence reduction as an environmental exposure and a priority under climate change—will help support these efforts, and better prepare communities for climate adaptation. Conceptualizing and prioritizing violence as a climate-related environmental variable may offer a paradigm shift in disease prevention, incorporating aspects of environment, climate, and prevention into public health policy and practice [112, 113].

## Conclusions

This manuscript provides some critical framing on conceptualizing local violence as an environmental variable, in the context of climate change, and to assess its impacts on perceived stress and health in concert with other climate-related environmental exposures (i.e., heat, air pollution).

Here, we considered the spatial and environmental dimensions of local violence, and offered insights on the measurement of local violence as it relates to perceived stress and stress pathways to health. Finally, we considered the multiple pathways through which local violence may act on both mental and physical health, and discussed important examples and implications for the consideration of local violence as a climate-related environmental exposure in both policy and practice.
